# Transcriptional Response of Honey Bee Larvae Infected with the Bacterial Pathogen *Paenibacillus larvae*

**DOI:** 10.1371/journal.pone.0065424

**Published:** 2013-06-06

**Authors:** Robert Scott Cornman, Dawn Lopez, Jay D. Evans

**Affiliations:** Bee Research Laboratory, Agricultural Research Service of the United States Department of Agriculture, Beltsville, Maryland, United States of America; Wageningen UR Livestock Research, The Netherlands

## Abstract

American foulbrood disease of honey bees is caused by the bacterium *Paenibacillus larvae*. Infection occurs *per os* in larvae and systemic infection requires a breaching of the host peritrophic matrix and midgut epithelium. Genetic variation exists for both bacterial virulence and host resistance, and a general immunity is achieved by larvae as they age, the basis of which has not been identified. To quickly identify a pool of candidate genes responsive to *P. larvae* infection, we sequenced transcripts from larvae inoculated with *P. larvae* at 12 hours post-emergence and incubated for 72 hours, and compared expression levels to a control cohort. We identified 75 genes with significantly higher expression and six genes with significantly lower expression. In addition to several antimicrobial peptides, two genes encoding peritrophic-matrix domains were also up-regulated. Extracellular matrix proteins, proteases/protease inhibitors, and members of the Osiris gene family were prevalent among differentially regulated genes. However, analysis of *Drosophila* homologs of differentially expressed genes revealed spatial and temporal patterns consistent with developmental asynchrony as a likely confounder of our results. We therefore used qPCR to measure the consistency of gene expression changes for a subset of differentially expressed genes. A replicate experiment sampled at both 48 and 72 hours post infection allowed further discrimination of genes likely to be involved in host response. The consistently responsive genes in our test set included a hymenopteran-specific protein tyrosine kinase, a hymenopteran specific serine endopeptidase, a cytochrome P450 (CYP9Q1), and a homolog of *trynity*, a zona pellucida domain protein. Of the known honey bee antimicrobial peptides, apidaecin was responsive at both time-points studied whereas hymenoptaecin was more consistent in its level of change between biological replicates and had the greatest increase in expression by RNA-seq analysis.

## Introduction

American foulbrood (AFB) is a bacterial infection of honey bee (*Apis mellifera*) larvae that is highly contagious and virulent [1]. The causative agent is *Paenibacillus larvae*, a gram-positive bacterium that establishes an initial infection in the midgut lumen after larvae consume spore-contaminated food. Systemic infection is achieved when vegetative cells breach the peritrophic membrane, a physical barrier to infection that is secreted by the anterior midgut, and then penetrate between epidermal cells [2]. Hosts succumb to septicemia during late larval or pupal development and the corpses are digested by vegetative bacterial cells and ultimately converted to dried scales containing millions of *P. larvae* spores. While antibiotics can be effective in controlling AFB, their use in some countries is curtailed or prohibited over concerns of honey contamination. Severely infected hives are usually destroyed to prevent the spread of spores.

While AFB remains a disease of economic concern, it is also a useful system for investigating genetic components of immunity in honey bees and the molecular interactions between host and pathogen that underlie pathogenesis. Honey bee larvae are only vulnerable to *P. larvae* by oral inoculation, and this susceptibility attenuates by approximately three days after hatching [1]. Adults are unaffected, narrowing the range of tissues and developmental stages that are relevant to disease progression. Larvae can be reared in the laboratory and inoculated with controlled doses of *P.* *larvae* (e.g., [2], [3], [4] (as well as other commensal or pathogenic bacteria), permitting a range of experimental manipulations. Importantly, genetically distinct strains of *P.* *larvae* with different levels of virulence have been identified [5], and colony-level variation in resistance has also been documented [6]. Hygienic removal of infected larvae by workers appears to be one component of resistance [7], [8], but other sources of heritable variation may exist as well. For example, the amount and type of antimicrobial proteins [9], [10] that are produced by larvae may vary genetically. Alternatively, there may be variation in the protein components of the peritrophic matrix, the first line of defense against germinating bacterial spores. Thus, this system is very tractable for investigating genetic, environmental, and genotype-by-environment components of honey bee immunity and has potentially broad application. For example, European foulbrood has a similar etiology to AFB but is initiated by an unrelated bacterium, *Melisococcus plutonus*. Whether there are overlapping mechanisms of host resistance and bacterial virulence for these two diseases is relevant both to apiculture and to the evolutionary ecology of insect immunity. *P. larvae* is also a useful indicator species for studies of pathogen suppression by commensal microorganisms [11] or environmental factors that suppress host immunity.

In this study, we estimated the expression of honey bee genes in control and infected larvae at 72 hours post infection (p.i.), using deep sequencing of mRNA. In addition to the expected up-regulation of genes encoding antimicrobial peptides, we identified two genes encoding peritrophic-matrix domains (Pfam 01607) that had increased expression whereas other genes encoding this domain were unchanged. All genes with the Osiris domain (Pfam DUF1676) [12] for which there was adequate read coverage were up-regulated, indicating that the unknown functions of these genes were broadly impacted by AFB. However, data available for *Drosophila* homologs of differentially expressed (DE) honey bee genes suggest that developmental asynchrony between treatment and control groups was a likely confounder of our results. To explore this issue, we used qPCR to measure the consistency of gene expression changes for a subset of the genes classified as DE, by comparing replicate cohorts at 72 hours and a single cohort at both 48 and 72 hours. These additional data showed that some host genes that were differentially represented in the sequencing pools are consistently responsive to mid-stage AFB infection whereas others are not. The consistently responsive genes in our test set included a hymenopteran-specific protein tyrosine kinase, a hymenopteran specific serine endopeptidase, a cytochrome P450 (CYP9Q1), and a homolog of *trynity*, a zona pellucida (ZP) domain protein. Of the known honey bee antimicrobial peptides, apidaecin was responsive at both time-points studied whereas hymenoptaecin was more consistent in its level of change between biological replicates and the most up-regulated gene by RNA-seq analysis.

## Materials and Methods

### *In-vitro* Rearing and Inoculation of Larvae

Newly hatched larvae were harvested in May of 2009 from a healthy colony of the U.S.D.A. Bee Research Laboratory apiary, in Beltsville, Maryland. Approximately 12-hour larvae were floated on the surface of a 250 ml drop of larval food in the center of a 15 ml petri dish using a grafting tool. The diet consisted of 125 µl Royal jelly, 37.5 µl of a 40% honey solution, 37.5 µl of a 40% glucose solution, and 2.5 mg yeast extract. The diet was inoculated with 50 µl of either sterile water or AFB inoculum. The AFB inoculum was prepared from a scale collected from a foulbrood-infested colony. The scale was suspended in 30 ml of sterile water, verified by microscopy, and heat-shocked to remove contaminating organisms. Spore counts were performed with a microscope and diluted to a concentration of 100 spores/µl. After grafting, plates were held at 34°C and high humidity. For both control and infected cohorts, eight larvae were collected at 72 hours p.i. and frozen at –80°C until RNA extraction. A replicate experiment was performed following the same protocol in September 2012, using an unrelated colony and a new AFB preparation, with eight individuals collected at 48 and 72 hours p.i. for each cohort.

### RNA Extraction and cDNA Synthesis

Total RNA was isolated from individual larvae following the RNAqueous 96-well plate extraction protocol, including the optional DNase step (Ambion). RNA quantity was determined with a Nanodrop 8000 spectrophotometer and integrity assessed by requiring a 260 nm/280 nm absorption ratio of ∼2. An additional DNase step was included prior to cDNA synthesis in an 11 µl reaction that consisted of 8 µl (1.5 µg) total RNA, 2 U DNAse1 with appropriate 1X buffer (Ambion), 20 U RNAseout (Invitrogen), 12- to 18-mer thymine oligonucleotides, random heptamer oligonucleotides, and 2 mM dNTP. The reaction was incubated at 37°C for one hour, followed by 75°C for 10 min, then cooled on ice and briefly centrifuged. The cDNA was diluted 1∶5 with ddH_2_O.

### RNA Preparation for Sequencing

For Illumina sequencing, we pooled 30 µl of RNA isolate from each of eight control larvae, and an equivalent amount from *P. larvae*-infected larvae. In addition to spectrophotometric analysis, we electrophoresed the extract in an agarose gel to confirm high molecular weight RNA. Ribosomal RNA was reduced with a RiboMinus Kit (Invitrogen) following the manufacturer’s protocol. Twelve nanograms of each pool was provided to the Institute for Genome Sciences, University of Maryland, Baltimore, for library preparation and sequencing on an Illumina GA-IIx instrument, following the manufacturer’s protocol. The sequencing center also confirmed RNA quality with an Agilent BioAnalyzer prior to library preparation.

### Quantitative Real-time PCR

Reactions consisted of 1.5 µg of template, 1 U Taq (Roche Applied Sciences), 1 mM dNTP mix, 2 mM MgCl_2_, 0.2 µM of each primer, 1X concentration SYBR-Green I dye (Applied Biosystems), and 10 nM fluorescein in a 25 µl volume with supplied reaction buffer. We used a Bio-Rad iCycler (Bio-Rad Corp) to perform an initial five-minute denaturing step at 95°C, followed by 40 cycles of 94°C for 20 s, 70°C for 30 s, 72°C for 1 min, and 78°C for 20 s. Fluorescence measurements were taken repeatedly during the 78°C step to minimize error due to fluorescence artifacts, and CT values for each reaction was based on the average of three technical replicates. The expected melt temperature was confirmed for each amplicon, and negative control reactions were run for each primer pair. The efficiency of each primer pair was estimated by dilution series [13] and the expression differential of each target was calculated by the ΔΔC_T_ method using the geometric mean of five reference genes [14]: ribosomal protein S5 (XM_624081), microsomal glutathione-S-transferase (XM_394313), ubiquitin (XM_003249801), UDP glucuronyltransferase (XM_392727), and alpha-tubulin (XM_391936). These reference genes were chosen because they had similar ΔC_T_ between infected and control cDNA pools and had read count differentials close to zero. qPCR primer sequences and amplicon characteristics are given in **File S1**. When no transcript was detected in a reaction, we usually assigned that sample a CT one cycle below the lowest detected, as it seems preferable to conclude that the transcript level was below detection limits rather than absent (all reactions for a gene for each replicate were on one plate). For one case in which most or all samples in a cohort had undetected transcript levels (see Results and Discussion), we simply excluded the gene for that cohort and time point.

In addition to honey bee targets, we also measured an mRNA marker expressed by *P. larvae*, the S18 ribosomal protein gene, which confirmed the presence or absence of transcriptionally active bacteria in treatment and control larvae, respectively (results not shown). While it is tempting to correlate the level of this marker with gene expression changes in individual larvae, i.e. to infer a dose-response effect, we do not believe there is a valid basis for interpreting those data. The dynamic nature of host-pathogen interactions and nonlinear or threshold effects make suspect any simple post-hoc comparison of transcript levels between the two species. For example, *P. larvae* markers and antimicrobial peptide production may be positively correlated early in infection, but the successful activity of antimicrobial peptides might neutralize the trend. A more sound approach would be to manipulate the inoculum dosage through serial dilution, but that is beyond the scope of the present study.

### Computation of Differentially Expressed Genes by RNA-seq

Sequence runs were deposited in the NCBI Short Read Archive under accessions SRX028146 and SRX028147. Illumina reads were trimmed to contiguous segments with a minimum Phred-equivalent quality score of 15, excepting at most one ambiguous base. Reads less than 50 bp after this trimming were discarded. Reads were then mapped to the *A. mellifera* reference gene set (version 4.5, http://hymenopteragenome.org/beebase/) using Bowtie [15]. We allowed up to two mismatches (ambiguous bases were considered mismatches) and used the ‘best’ option to identify the most likely source of each read.

To calculate the ratio of read counts in the treatment sample to those in the control sample, we added a pseudocount of 0.5 to the total number of reads mapped to each transcript so that all ratios were defined (i.e., to remove zero values). We used edgeR [16] to perform Fisher’s exact test for each transcript to determine if the proportion of reads in the treatment sample differed significantly from that of all other transcripts combined, using the Benjamini-Hochberg correction for multiple tests and an α of 0.0001. In addition to a significant test statistic, only transcripts with at least a two-fold difference and at least 25 reads mapped in either sample were classified as differentially expressed. We added these more conservative criteria because biological replicates within each treatment class were pooled for sequencing and thus among-replicate variation cannot be estimated.

## Results and Discussion

Illumina sequencing of the control and AFB-infected pools resulted in 20,731,161 and 19,646,086 quality-trimmed reads, respectively. The mean read lengths were reduced after quality trimming from 75 bp to 74.6 and 74.0 bp, respectively. Despite a ribosomal depletion step and polyT-priming (in addition to random heptamers) during cDNA synthesis, the enrichment of mRNA relative to ribosomal RNA was unexpectedly low. The number of quality-filtered reads that mapped to honey bee transcripts was 688,125 for the control pool and 573,223 for the AFB-infected pool, clearly limiting our ability to detect differential expression of weakly expressed genes. Nonetheless, 3,993 of 11,736 reference genes (34%) had a maximum read count of 25 or more in either library, despite the narrow life-history window examined. The data therefore provide a large-scale snapshot of transcriptional responses to infection by *P. larvae*.

**Figure 1:**
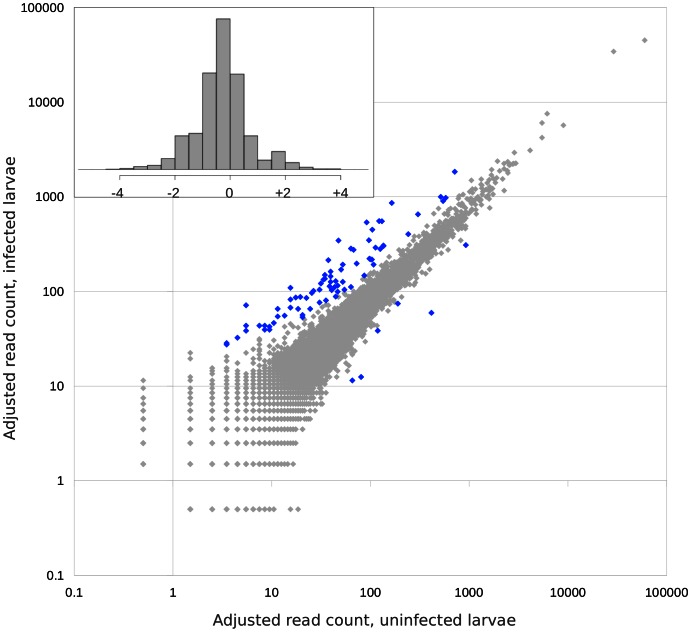
Relative abundance of honey bee transcripts in treated versus control larvae 72 hours post-infection. The scatterplot shows the count of reads mapping to each transcript with control values on the horizontal axis and infected values on the vertical axis. All values include a pseudocount of 0.5 reads, see Materials and Methods. Inset shows a histogram of log2 differential abundance, adjusted for library size.

A scatterplot of the number of reads mapped to each *A. mellifera* gene is shown in **Figure 1**. Reads were summed over all annotated transcripts of each gene and a pseudocount of 0.5 was added to all detected genes to eliminate any pairing of zero and non-zero values (for plotting in figures and the computation of log2 differential expression, but not for the Fisher’s exact test mentioned below). Seventy-five genes were classified as being more highly expressed in the *P. larvae*-infected cohort and only six were classified as having higher expression in controls (i.e., at least a two-fold difference in read count and a Benjamini-Hochberg adjusted p-value <1e-5 for Fisher’s exact test). Genes classified as differentially expressed by these criteria are detailed in **File S2**. Read count differentials were generally modest, with almost all differences less than 10-fold.

### Expression Ratios of Genes Related to Immunity and to the Peritrophic Matrix

In addition to the honey bee transcriptome as a whole, we also looked at two sets of genes that we considered *a priori* to be candidates for differential regulation during *P. larvae* infection. These were (1) the honey bee immune genes annotated in [9] and listed by category in Table 1 of [17], and (2) genes contributing structurally to the peritrophic matrix, a physical barrier of chitin and protein surrounding ingested material in the midgut lumen, which must be breached prior to systemic infection [2]. The total protein content of the insect peritrophic matrix is not well known, but includes mucins, proteases, and chitin-binding proteins (peritrophins) (reviewed by [18], [19], [20]). We restricted our analysis to the 42 honey bee genes we identified that encode the CBM-14/peritrophin-A protein domain (Pfam 01607), a protein domain strongly associated with the peritrophic matrix [18], [19], [20], although not necessarily exclusively so.

**Table 1:** Assignment of honey bee genes to *Drosophila* developmental expression clusters.

*Drosophila* developmental gene expression cluster number	Number of differentially expressed honey bee genes assigned to cluster
7	28
Not clustered	8
12	6
29	6
25	5
4	3
0	2
14	2
20	2
9	1
10	1
11	1
13	1
22	1
27	1
31	1

Differentially expressed honey bee genes were assigned to the *Drosophila* developmental expression cluster of their closest *Drosophila* homolog (threshold TBLASTX E-value of 1.0E-10). Developmental expression clusters were delineated by the modENCODE project [30] and are available from Flybase [37].

**Figure 2:**
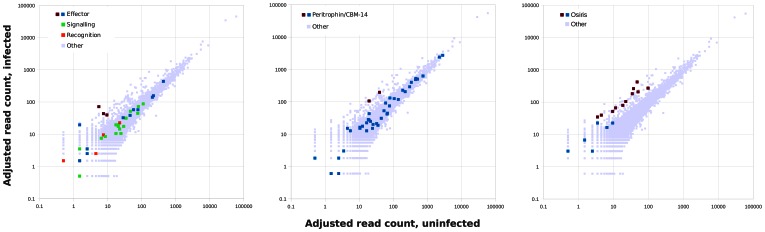
Differential abundance of specific classes of transcripts. All read counts include a pseudocount of 0.5 reads, see Materials and Methods. A) Immune genes annotated by [9] and categorized in Table 1 of [17]. B) Genes encoding peritrophin-A/CBM-14 domains (Pfam 01607). C) Genes encoding Osiris domains (Pfam DUF1676) [12].

**Figure 2a** shows the pattern of read mappings to 38 honey bee immune genes. Several effector peptide transcripts were up-regulated, but recognition and signaling genes were not visibly changed as a group. Hymenoptaecin (NM_001011615), apidaecin (XM_003249457), and defensin1 (NM_001011616) were the antimicrobial peptides significantly up-regulated. The antimicrobial peptide abaecin (NM_001011617) had a comparable log ratio to the other up-regulated transcripts but the minimum read count and p-value fell short of our conservative thresholds (19 reads in the infected pool versus 1 in the control pool, p = 0.00056). The lack of response in recognition and signaling transcript contrasts with previous work that did show changes in these classes [9]. Moreover, we observed no increase in other immune factors such as lysozyme or prophenyloxidase that have been found more abundant in 5-day larvae challenged with *P. larvae* [4]. Hypotheses that can be tested with further experiments are that resistant *A. mellifera* lineages have higher constitutive expression of recognition/signaling components that induce these microbial peptides, or achieve greater induction of effectors per unit of recognition/signaling transcript.

**Figure 2b** shows read mappings to the 42 candidate peritrophic-matrix genes. Only two were significantly up-regulated (XM_393988 and XM_003250167), each showing an approximately five-fold increase in expression that is quite distinct from the trend for other peritrophins. These genes appear to be specifically up-regulated compared with other family members, as opposed to being merely the outliers of a random sample of the transcriptome as a whole. XM_393988 also encodes an extensive collagen repeat that might help strengthen the peritrophic matrix. *In situ* hybridization studies are needed to confirm expression of these genes in the midgut epidermal cells responsible for peritrophic secretions.

### Enriched Categories among Differentially Expressed Genes as a Whole

Categories that were prominent among genes with increased expression (**File S2**) include proteases and protease inhibitors, extracellular matrix (ECM) components such as mucins and lectins, cuticle-associated proteins, and ZP-domain proteins including homologs of the *Drosophila* genes *dusky*, *dusky-like*, *miniature*, *neyo*, and *trynity* [21]. *dusky*, *dusky-like*, and *miniature* are involved in cuticle attachment to the epidermis [22] and ZP-domain proteins generally are involved in ECM assembly [23]. Proteases and their inhibitors are involved in diverse processes including ECM remodeling [24] and insect immune response [25]. Several genes encoding low-complexity proteins with signal peptides (XM_393452, NM_001142637, and XM_625286) are also likely to be ECM components, based on the presence of glycine-rich repeats [26] and/or YLP-like motifs [27]. Also up-regulated were three E-class cytochrome p450 genes, a common class of oxidizing enzymes in animals that have diverse roles including the metabolism of toxins [28].

The small number of genes with significantly lower expression in the treatment group by our criteria was dominated by those encoding putative ECM proteins. Two genes, XM_392861 and XM_001120541, encode homologs of the predominant cuticular protein family of arthropods (Pfam domain 00379) [26]. XM_001123255 and XM_001122443 encode low-complexity proteins with amino-acid compositions characteristic of cuticular proteins, i.e. rich in alanine, proline, and tyrosine [26], whereas XM_003249534 has YLP-like repeats that are characteristic of certain ECM proteins including some in cuticle [27]. All of these predicted proteins have signal peptides for translation into the secretory pathway.

Remarkably, 11 up-regulated genes were homologues of the Osiris gene family [12], a conserved family of unknown function in insects (Pfam domain DUF1676) that have signal peptides and transmembrane domains and thus are likely to have extracellular activities. Most Osiris genes in *Drosophila* show spikes of high to very high expression at various developmental stages and are prominently expressed in gut tissues [29], [30]. We scanned the predicted proteome of *A. mellifera* with Hmmer [31] and identified a total of 17 putative Osiris genes in honey bee, the read counts for which are shown in **Figure 2c**. Remarkably, all 17 genes have higher normalized read counts in the infected sample than in control, although the total counts for six of these genes are negligible. It appears that transcript abundance of this gene family is broadly responsive to AFB infection, in contrast to the peritrophic-matrix and immune regulartory/signaling genes examined above.

As 69 of 81 differentially expressed genes (85.2%) had TBLASTX matches to *D. melanogaster* with an E-value <1e-10, we performed a gene ontology (GO) enrichment analysis using the GO annotations for each putative *Drosophila* homolog [32]. The cellular compartment terms “integral to plasma membrane” (GO:0005887) and “extracellular” (GO:0005576) were significantly enriched, as was the molecular function term “structural component of chitin-based cuticle” (GO:0005214) (p<0.05 by Fisher’s Exact Test, adjusted for multiple tests using a false-discovery-rate method). These results are consistent with and reinforce our descriptive interpretation of functional categories associated with differentially expressed honey bee genes. We also submitted these homologs to the DAVID functional annotation tool [33] using the “medium stringency” setting, the output of which is shown in **File S3**. Enriched annotation clusters that were identified by DAVID also corroborated the descriptions above: proteases, cuticle-related proteins, and other extracellular matrix proteins such as lectins were identified as enriched, as well as redox activity (cytochrome p450 genes) and epidermal growth factor-like domains. Only the cuticle-related/ZP-domain annotation cluster was significant when corrections for multiple tests were considered (**File S3**), however.

*Prima facie*, many of the up-regulated honey bee genes have inferred functions consistent with roles in the active repair of damaged epithelium, or, speculatively, the immobilization of invading pathogens via melanization. Furthermore, ECM proteins are common binding targets of pathogenic bacteria [34], [35] and could conceivably experience altered expression during infection. However, the prevalence of genes that normally have strong peaks of expression during development and that are not expected to be expressed in the midgut (e.g., cuticle-associated genes), particularly among the few genes with significantly decreased expression, suggest an alternative hypothesis. At least some of the genes may be biomarkers of developmental asynchrony between treatment and control cohorts, rather than directly responsive to infection. Delay or failure of normal developmental programs might well be expected in infected larvae 72 hours post infection, such that the observed changes in gene expression would reflect a mix of direct and indirect effects.

To better evaluate the relative contributions of disease-induced responses versus developmental asynchrony, we analyzed the spatial and temporal expression patterns of the *Drosophila* homologs of differentially expressed honey bee genes. In so doing, we assumed that the developmental patterns of gene expression in fruitfly are suitable models for those in honey bee. The goals of this analysis were 1) to determine whether *Drosophila* homologs of differentially expressed honey-bee genes are normally expressed in the larval midgut, and thus potential constituents of the midgut epidermis or peritrophic matrix; and 2) to determine whether these homologs were disproportionately represented in modENCODE gene expression clusters [30] that have a strong, narrow peak during mid-larval development, which would indicate diverging developmental rates between cohorts at 72 hours p.i.

**Figure 3:**
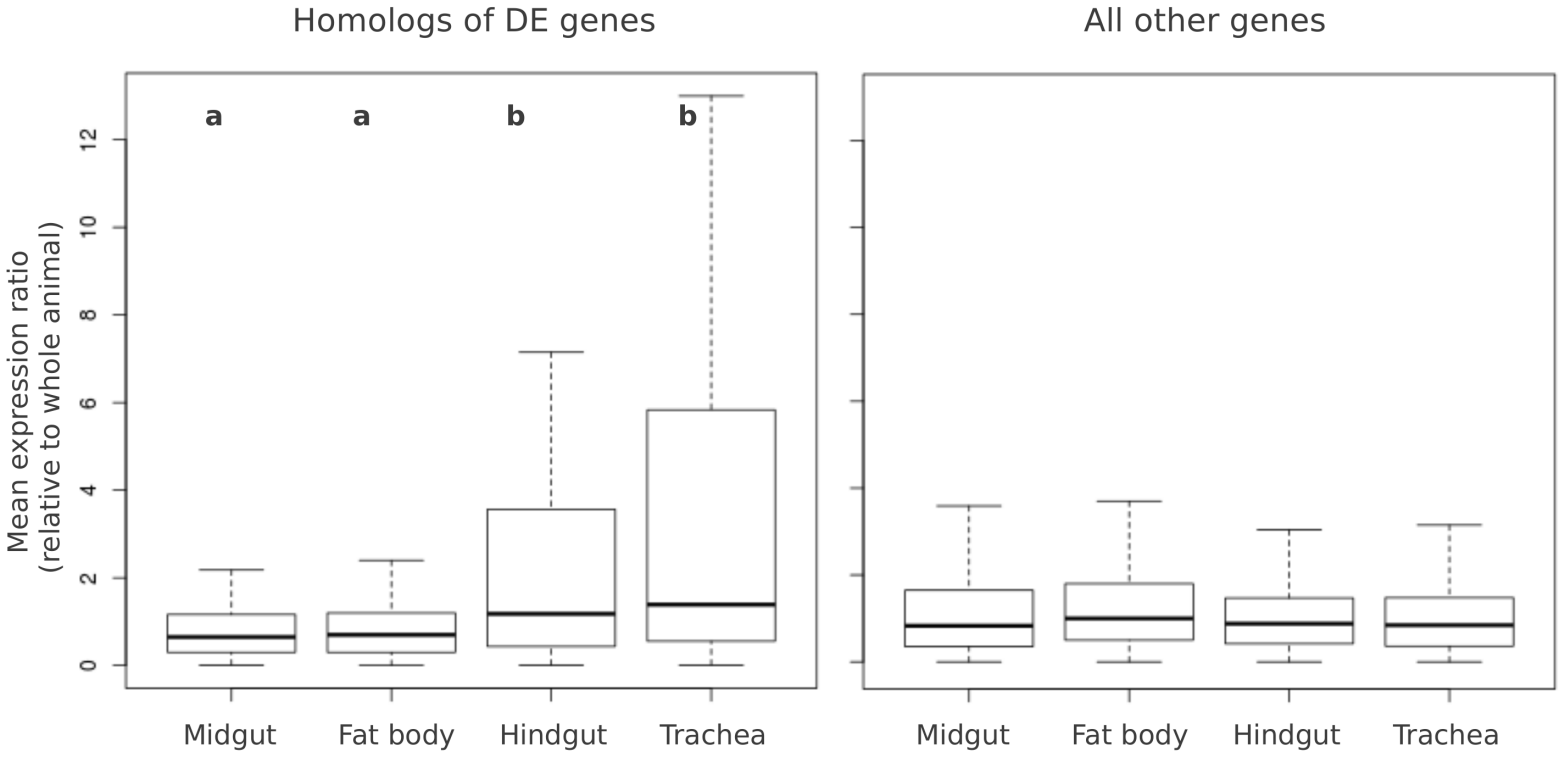
Boxplot of mean expression of *Drosophila* genes in four larval tissues, relative to the value measured for the whole adult animal. Raw data are from the FlyAtlas project [29]. The left panel plots the mean ratios for *Drosophila* homologs of honey bee DE genes; the right panel plots the mean ratios for all other *Drosophila* genes. For *Drosophila* homologs of honey bee DE genes, mean expression was significantly higher in hindgut and trachea (ANOVA P<0.0001, pairwise P<0.01) than midgut and fat body. No significant difference was observed among tissues for all other *Drosophila* genes. We included all microarray probes associated with each gene, and removed outliers values (values greater than 12).

First, we used microarray data for four larval tissues available from Fly Atlas [29]. The microarray data showed that these homologs have greater expression during normal development in the hindgut and trachea compared with fat body and the midgut (**Figure 3**). Since the midgut is where *P. larvae* infection initiates [2] and is unconnected with the hindgut during most of larval development [36], the lack of midgut specificity in the *Drosophila* homologs of differentially expressed genes casts doubt on the notion that they are being induced locally in response to tissue damage.

**Figure 4:**
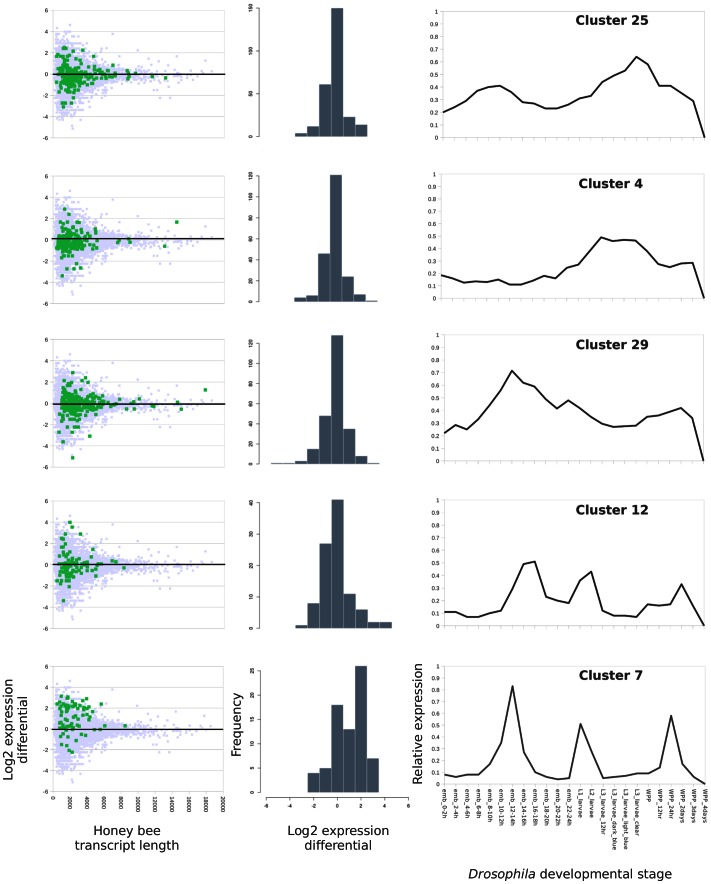
Relative expression of honey bee genes, binned by *Drosophila* expression cluster. For each developmental gene-expression cluster defined by the modENCODE project [30], the closest honey bee homolog of each *Drosophila* gene in that cluster was identified by BLAST and binned accordingly. The left panel shows the log2 relative abundance of each honey bee gene binned into the given cluster (green squares), together with all other honey bee genes (grey squares). The values are plotted as a function of transcript length (X axis), which contributes to the variance in differential expression estimates and could potentially co-vary with expression cluster. The middle panel shows a histogram of log2 abundance for all honey bee genes mapped to the cluster. The right panel shows the median relative expression (scaled from 0 to 1) of *Drosophila* genes in each expression cluster during embryonic, larval, and pupal developmental stages, thereby illustrating the characteristic pattern of gene expression in each cluster.

We then downloaded whole-organism microarray data for defined developmental stages of *Drosophila*, available from modENCODE [30] and viewable in Flybase [37]. Inspection of these expression data revealed a frequent pattern in which homologs of differentially expressed honey-bee genes have rapidly increasing expression early in larval development, achieve high maxima in mid-larval stages, and then rapidly decline in late larval development. Roy and colleagues [30] defined over 30 developmental gene-expression clusters from these data, which we used to bin differentially expressed genes (**Table 1**). Expression cluster 7 was highly represented among these genes (28 of the 69 genes with *Drosophila* homologs, or 40.6%), and has a single strong peak at the L1 stage of *Drosophila* development (**Fig. 4** bottom-right panel). Moreover, we can reverse the direction of the comparison and identify all *A. mellifera* homologs (best TBLASTX match with an expectation threshold of 1E-10) of *D. melanogaster* genes in each expression cluster and investigate the pattern of change in each group, rather than just examining significantly differentially expressed genes. Plotting the expression differentials of honey bee homologs to *Drosophila* genes for each of the most-frequent expression clusters in **Table 1** (clusters 4, 7, 12, 25, and 29), cluster 7 displays a clearly bimodal pattern of differential expression with the primary mode at a log2 difference of approximately 2 and a secondary mode near zero (**Fig. 4**). The mean log2 differential in infected bees for cluster 7 genes was 1.06.

One interpretation of these data is that the 72-hour sampling point for honey bee larvae is comparable to the *Drosophila* L2 stage, and the energy depletion [4] and tissue damage [2] associated with infection has delayed some developmental programs that are normally elaborated during the honey-bee analog of the L1–L2 interval. This hypothesis explains the apparent increased expression of those genes at that end of the interval, and would also imply that genes with expression peaks after this interval would appear to be down-regulated. This in fact appears to be the situation for genes assigned to expression cluster 12: this cluster also shows a strong peak of larval expression in *Drosophila* that begins at the L1 stage in *Drosophila* but does not reach a maximum until the L2 stage. The smaller magnitude of the shift in relative expression that is evident in the scatterplot and histogram is consistent with the smaller differences between L1 and L2 expression in expression cluster 12. The other three expression clusters shown do not have narrow peaks of expression during larval development and the distribution of log2 differential abundance mirrors that for honey bee genes as a whole. The reasonableness of comparing 72-hour honey bee larvae with the *Drosophila* L2 stage, which terminates around 48 h post emergence [38], is buttressed by a proteomic comparison of honey bee larval development [4] that identified expression patterns that are conserved with *Drosophila* and by the fact that the larval period is longer in honey bee [39].

Collectively, the results of our bioinformatic comparison to the *Drosophila* model revealed temporal and spatial patterns of expression that are not consistent with a simple model of gene regulation in response to the invading pathogen. Rather, there is a significant ascertainment bias in our results that favored detection of genes with strong expression peaks, confounding the identification of disease-response pathways. Interestingly, a number of gene-expression studies of adaptive evolution in insects have identified cuticular proteins as being among the most significantly differentially expressed genes between two phenotypic groups [40], [41], [42]. Given that many cuticular protein genes have narrow windows of very high peak expression associated with molts [43], [44], this ontological group seems particularly susceptible to being detected as differentially expressed whenever developmental asynchrony occurs between cohorts. Indeed, the adaptive phenotypes studied in the cited papers are largely developmental in nature, such that biomarkers of developmental asynchrony between phenotypic groups should be expected. We suggest that differential expression of cuticular protein genes, and other genes with comparably sharp peaks of expression, while replicable, may not be causally linked to phenotypes being investigated. Of course, the difficulty of distinguishing primary from secondary effects of an experimental treatment is common to many investigations; we raise the issue only as a precautionary note, as there are ever-increasing genomic resources for arthropod models that can help disentangle direct from indirect responses, as we have tried to do here. Visible markers of developmental stage are often used for this purpose, but the accessibility of such markers differs greatly among organisms and mRNA or protein markers of developmental events are more likely to be broadly applicable.

### qPCR-based Identification of Genes Consistently Responsive to Foulbrood Infection

Our RNA-seq approach was designed to quickly identify a set of candidate AFB-responsive genes, and indeed captured the expected response in antimicrobial peptide production, providing independent corroboration of our genome-scale approach. Although the set of differentially expressed genes was relatively small and dominated by only a few protein classes (e.g., peptidases, extracellular matrix proteins, Osiris domain proteins), the comparisons with *Drosophila* described above indicate that at least some of these genes are only secondarily responsive, due to developmental asynchrony. We therefore tailored our qPCR validation of these candidates to identify genes that are consistently responsive across diverse biological replicates as well as over a broader time period, 48–72 hours p.i. We consider genes that meet these criteria to be much stronger candidates for being directly up-regulated due to AFB infection and the highest priority for additional quantitative and functional studies. We initially chose 14 genes to investigate that represent a diversity of functional domains/ontological groups (**Table 2**). Note that we were not able to achieve sufficiently high qPCR efficiency for GB14309 (XM_393316) to have confidence in numerical estimates of relative expression (**File S1**), but we nonetheless included it for comparative purposes across replicates and time points.

**Table 2:** Relative expression of fourteen honey bee genes as measured by RNA-seq and qPCR.

Accession	Description	Expression cluster	RNA-Seq,72 h	qPCR of sequencedpool, 72 h	qPCR of replicate cohort (n = 8), 72 h	qPCR of replicate cohort (n = 8), 48 h
NM_001011615	Hymenoptaecin	n/a	3.98	2.97	2.97	−1.19
XM_001121961	Osiris domain	7	3.14	0.95	not detected	not detected
XM_393316	Serine peptidase	7	3.10	1.67	3.73	3.13*
XM_625174	Uncharacterized	7	2.84	2.32	5.31	0.482*
XM_003249457	Apidaecin	n/a	2.82	1.67	3.85	2.27***
XM_001121985	Osiris domain	7	2.80	1.67	2.86*	−8.23
XM_393849	Tyrosine receptor kinase/cadherin	29	2.63	2.46	1.99*	3.54***
XM_394451	trynity (ZP-domain)	7	2.46	1.56	3.75	2.31**
XM_003250167	Peritrophin/chitin-binding domain		2.45	3.53	4.88*	−3.18*
XM_393988	Peritrophin/chitin-binding domain, collagen domain	20	2.32	3.52	5.99**	−0.09
XM_391992	Myosin-like	none	1.39	1.61	−0.76	−0.46
XM_391948	MARVL-domain protein	29	1.05	1.90	−0.06	9.50*
XM_393971	Cytochrome P450 CYP9Q1	none	1.02	0.83	3.18**	4.15***
XM_392861	CPR family cuticular protein	12	−2.23	−0.56	1.39	−1.82

* P<0.1, **P<0.01, ***P<0.001.

The fourteen genes are a subset of the 81 genes identified as differentially expressed by RNA-seq. The first two columns of data are technical replicates of the same biological sample, whereas the latter two columns represent a different cohort and include an additional time point. P-values are indicated for the two-sided t-test of equal means ΔC_t_ in treatment and control groups, where ΔC_t_ is the approximate expression differential on a log2 scale relative to the geometric mean of five reference genes. T-tests were performed on the log2 values because the linear values deviate strongly from a normal distribution. Expression cluster is that assigned to the best *Drosophila melanogaster* homolog of each gene by [30]; “n/a” indicates that no *Drosophila* homolog exists and “none” indicates that the *Drosophila* homolog was left unclustered by [30].

**Table 2** shows the log2 expression differential between the original AFB-infected and control pools determined by RNA-seq, the differential measured by qPCR for the same cDNA, and the qPCR differential for a second biological replicate of the original experiment. The replicate experiment was conducted with a different hive, a different spore isolate, and in a different year. In this replicate, we measured the means of eight treated larvae and eight control larvae rather than pooled samples so we could measure individual variation. The Pearson correlation coefficient between mean log2 expression differentials (72 hours p.i.) in the two experiments was 0.72.

Of the 14 genes we investigated by qPCR, 12 had an increase of 50% or more in mean expression in the second infected cohort at 72 hours relative to control. However, only five genes had increased expression at both 48 and 72 hours p.i.: apidaecin, a tyrosine receptor kinase, a cytochrome P450 (CYP9Q1), a serine endopeptidase, and a zona pellucida (ZP) domain protein. For the Osiris gene XM_001121961, we were not able to quantify the relative change in expression in the replicate experiment at 48 or 72 hours p.i., because transcripts were not detected in any individual of the infected group nor in most members of the control group, despite the high estimated efficiency of our primers. With respect to our hypothesis of developmental delay, both Osiris genes and an uncharacterized gene (XM_625174) that mapped to *Drosophila* expression cluster 7 were inconsistent across replicates and time points, suggesting that they are not directly responsive to AFB infection. However, the tyrosine receptor kinase (which also includes a cadherin domain) and the serine endopeptidase noted above map to expression cluster 7 as well, suggesting that at least some genes mapping to this cluster may in fact be directly responsive, and there is no reason these two explanations should be mutually exclusive.

**Table 2** also gives the P-value for equal means of relative gene expression between treated and control larvae (two-sided t-test, unadjusted for multiple tests). Despite the small sample size (N = 8) and hyperdispersion of gene-expression data generally [45], CYP9Q1 and the tyrosine receptor kinase were significantly differentially expressed at both time points and the endopeptidase and apidaecin were significantly different at 48 hours. Interestingly, all five of the genes with increased expression at both time points had lower P-values for the 48-hour comparison, suggesting a generally stronger response at this time period.

### Conclusion

We used an RNA-seq approach to efficiently identify genes that have increased expression in honey bee larvae during AFB infection, and performed additional validation with qPCR. As expected, known anti-microbial peptides were up-regulated, the most consistent of which overall was apidaecin although hymenoptaecin had the highest increase in expression by RNA-seq. The increase in hymenoptaecin expression was virtually identical between the two replicates whereas apidaecin was quite different in magnitude. We also found that only two of 42 putative peritrophins had increased expression by RNA-seq and these were not increased at both 48 and 72 h, indicating that peritrophic matrix components are not broadly up-regulated in response to AFB infection. It remains possible that the timing or magnitude of constituitive expression of peritrophic matrix components contributes to variation in resistance among lineages.

In addition to immunity and peritrophin-domain genes that were considered of interest *a priori*, several other functional classes were prominent among differentially expressed genes. These included proteins potentially associated with the extracellular matrix of the damaged midgut epithelium, such as peptidases and structural components of ECMs. However, the up-regulation and down-regulation of a number of cuticular proteins, which are not expected to be expressed in the midgut epithelium [26], the disproportionate representation of homologs of *Drosophila* gene expression cluster 7, and the lack of midgut specificity of *Drosophila* homologs of differentially expressed genes all indicate a sizeable contribution of developmental asynchrony by 72 hours p.i. Using qPCR to validate genes that are consistently up-regulated across diverse biological replicates and at both 48 and 72 hours p.i., we were able to narrow and strengthen the list of AFB-responsive genes, providing new candidates for quantitative and functional analysis.

Our results present immediate routes for follow-up analysis, including fine-resolution temporal expression analysis, *in situ* hybridization, and RNAi, as well as protein-level analysis with specific antibodies or mass-spectrometry proteomics. The potential role of CYP9Q1 is particularly intriguing, as the protein has been shown to contribute to the detoxification of pesticides [46], begging the question of what its non-anthropogenic targets might be. The receptor tyrosine kinase and endopeptidase we identified have strong BLAST matches only to other hymenopterans outside of the conserved domains, so relatively little can be inferred regarding their function from model organisms. In contrast, the ZP-domain protein encoded by *trynity* is well-conserved in insects; in *Drosophila* it has roles in embryonic morphology and is expressed by the larval gut. It is part of a larger superfamily of ZP-domain proteins [21] that includes *neyo*, *dusky*, *dusky-like*, and *miniature*, all of which were also significantly up-regulated in the RNA-seq data (**File S2**), but all of which are also in *Drosophila* gene expression cluster 7. An essential task of follow-up work is to determine whether these genes are directly responsive to AFB infection, perhaps functioning in wound repair. A similar onus derives from the observation of so many members of the Osiris gene family among up-regulated genes.

Our sequencing and qPCR analyses of gene expression are based on whole larvae, predominantly at 72 hours p.i. with comparative data at 48 hours p.i. In comparison, [4] used proteomic analysis of hemolymph to investigate the progress of AFB infection in larvae at 5 days p.i. In addition to an increase in hymenoptaecin with infection, they also detected increases in two other immune factors, lysozyme and phenoloxidase, that were not differentially abundant in our RNA-seq data (log2 differences of −0.27 and −0.24, respectively). These different findings could be related to the time points and tissues chosen for each survey, but they also highlight how transcriptomics and proteomics can glean distinct, hopefully complementary, insights into the same biological phenomenon. The abundance of a protein can substantially lag the production of its mRNA, and dynamic processes of post-transcriptional and post-translational regulation can uncouple the abundances of the two molecules [47]. For example, [4] argued that the increase in phenoloxidase was at least partly attributable to increased activation of prophenoloxidase rather than new transcription. Interestingly, [4] also detected a compelling metabolic signature of starvation in AFB-infected larvae that was not directly evident in our whole-animal expression analysis; conversely, molecular events occurring in the midgut would not have been directly accessible by their methods.

## Supporting Information

File S1 **Sequence and efficiencies of qPCR primers used in this study.**
(XLS)

File S2 **Summary of honey bee genes identified as differentially expressed in this study.**
(XLS)

File S3 **Annotation clusters among differentially expressed genes, identified by the DAVID annotation tool** [**33**] **using the best** ***Drosophila*** **homolog as proxy.**
(XLSX)

## References

[1] Shimanuki H (1990) Bacteria. In: Morse RA, Nowogrodski R, editors. Honey Bee Pests, Predators, and Diseases. 2nd ed: Cornell University Press. 27–47.

[2] Yue D, Nordhoff M, Wieler LH, Genersch E (2008) Fluorescence in situ hybridization (FISH) analysis of the interactions between honeybee larvae and *Paenibacillus larvae*, the causative agent of American foulbrood of honeybees (*Apis mellifera*). Environmental Microbiology 10: 1612–1620. 18331334 10.1111/j.1462-2920.2008.01579.x

[3] Evans JD (2004) Transcriptional immune responses by honey bee larvae during invasion by the bacterial pathogen, *Paenibacillus larvae* . Journal of Invertebrate Pathology 85: 105–111. 15050840 10.1016/j.jip.2004.02.004

[4] Chan QWT, Melathopoulos AP, Pernal SF, Foster LJ (2009) The innate immune and systemic response in honey bees to a bacterial pathogen, *Paenibacillus larvae* . BMC Genomics 10: 387. 19695106 10.1186/1471-2164-10-387 PMC2907699

[5] Genersch E, Ashiralieva A, Fries I (2005) Strain- and genotype-specific differences in virulence of *Paenibacillus larvae* subsp. *larvae*, a bacterial pathogen causing American foulbrood disease in honeybees. Applied and Environmental Microbiology 71: 7551–7555. 16269801 10.1128/AEM.71.11.7551-7555.2005 PMC1287710

[6] Evans JD, Pettis JS (2005) Colony-level impacts of immune responsiveness in honey bees, *Apis mellifera* . Evolution 59: 2270–2274. 16405170 10.1111/j.0014-3820.2005.tb00935.x

[7] Spivak M, Gilliam M (1998) Hygienic behaviour of honey bees and its application for control of brood diseases and varroa Part I. Hygienic behaviour and resistance to American foulbrood. Bee World 79: 124–134.

[8] Rauch S, Ashiralieva A, Hedtke K, Genersch E (2009) Negative correlation between individual-insect-level virulence and colony-level virulence of *Paenibacillus larvae*, the etiological agent of American foulbrood of honeybees. Applied and Environmental Microbiology 75: 3344–3347. 19304833 10.1128/AEM.02839-08 PMC2681656

[9] Evans JD, Aronstein K, Chen YP, Hetru C, Imler J-L, et al (2006) Immune pathways and defence mechanisms in honey bees *Apis mellifera* . Insect Molecular Biology 15: 645–656. 17069638 10.1111/j.1365-2583.2006.00682.x PMC1847501

[10] Schmid-Hempel P (2005) Evolutionary ecology of insect immune defenses. Annual Review of Entomology 50: 529–551. 10.1146/annurev.ento.50.071803.130420 15471530

[11] Evans JD, Lopez DL (2004) Bacterial probiotics induce an immune response in the honey bee (Hymenoptera: Apidae). Journal of Economic Entomology 97: 752–756. 15279248 10.1603/0022-0493(2004)097[0752:bpiair]2.0.co;2

[12] Dorer DR, Rudnick JA, Moriyama EN, Christensen AC (2003) A family of genes clustered at the Triplo-lethal locus of *Drosophila melanogaster* has an unusual evolutionary history and significant synteny with *Anopheles gambiae* . Genetics 165: 613–621. 14573474 10.1093/genetics/165.2.613 PMC1462804

[13] Schmittgen TD, Livak KJ (2008) Analyzing real-time PCR data by the comparative CT method. Nature Protocols 3: 1101–1108. 18546601 10.1038/nprot.2008.73

[14] Vandesompele J, De Preter K, Pattyn F, Poppe B, Van Roy N, et al (2002) Accurate normalization of real-time quantitative RT-PCR data by geometric averaging of multiple internal control genes. Genome Biology 3: R34. 10.1186/gb-2002-3-7-research0034 PMC126239 12184808

[15] Langmead B, Trapnell C, Pop M, Salzberg SL (2009) Ultrafast and memory-efficient alignment of short DNA sequences to the human genome. Genome Biology 10: R25. 19261174 10.1186/gb-2009-10-3-r25 PMC2690996

[16] Robinson MD, McCarthy DJ, Smyth GK (2010) Bioconductor package for differential expression analysis of digital gene expression data. Bioinformatics 26: 139–140. 19910308 10.1093/bioinformatics/btp616 PMC2796818

[17] Evans JD (2006) Beepath: an ordered quantitative-PCR array for exploring honey bee immunity and disease. Journal of Invertebrate Pathology 93: 135–139. 16737710 10.1016/j.jip.2006.04.004

[18] Lehane MJ (1997) Peritrophic matrix structure and function. Annual Review of Entomology 42: 525–550. 10.1146/annurev.ento.42.1.525 15012322

[19] Terra WR (2001) The origin and functions of the insect peritrophic membrane and peritrophic gel. Archives of Insect Biochemistry and Physiology 42: 47–61. 10.1002/arch.1036 11376452

[20] Hegedus D, Erlandson M, Gillott C, Toprak U (2009) New insights into peritrophic matrix synthesis, architecture, and function. Annual Review of Entomology 54: 285–302. 10.1146/annurev.ento.54.110807.090559 19067633

[21] Jazwinska A, Affolter M (2004) A family of genes encoding zona pellucida (ZP) domain proteins is expressed in various epithelial tissues during *Drosophila* embryogenesis. Gene Expression Patterns 4: 413–421. 15183308 10.1016/j.modgep.2004.01.003

[22] Roch F, Alonso CR, Akam M (2003) *Drosophila miniature* and *dusky* encode ZP proteins required for cytoskeletal reorganisation during wing morphogenesis. Journal of Cell Science 116: 1199–1207. 12615963 10.1242/jcs.00298

[23] Jovine L, Qi H, Williams Z, Litscher E, Wassarman PM (2002) The ZP domain is a conserved module for polymerization of extracellular proteins. Nature Cell Biology 4: 457–461. 12021773 10.1038/ncb802

[24] Stamenkovic I (2003) Extracellular matrix remodelling: the role of matrix metalloproteinases. Journal of Pathology 4: 448–464. 10.1002/path.1400 12845612

[25] De Gregorio E, Spellman P, Rubin G, Lemaitre B (2001) Genome-wide analysis of the *Drosophila* immune reponse by using oligonucleotide microarrays. Proc Natl Acad Sci U S A 22: 12590–12595. 10.1073/pnas.221458698 PMC60098 11606746

[26] Willis JH (2010) Structural cuticular proteins from arthropods: annotation, nomenclature, and sequence characteristics in the genomics era. Insect Biochemistry Molecular Biology 40: 189–204. 20171281 10.1016/j.ibmb.2010.02.001 PMC2872936

[27] Cornman RS (2010) The distribution of GYR- and YLP-like motifs in *Drosophila* suggests a general role in cuticle assembly and other protein-protein interactions. PLoS One 5: e12536. 20824096 10.1371/journal.pone.0012536 PMC2932725

[28] de Montellano P, editor (2004) Cytochrome P450: structure, mechanism, and biochemistry: Springer.

[29] Chintapalli VR, Wang J, Dow JA (2007) Using FlyAtlas to identify better *Drosophila melanogaster* models of human disease. Nature Genetics 39: 715–720. 17534367 10.1038/ng2049

[30] Roy S, Ernst J, Kharchenko PV, Kheradpour P, Negre N, et al (2010) Identification of functional elements and regulatory circuits by *Drosophila* modENCODE. Science 330: 1787–1797. 21177974 10.1126/science.1198374 PMC3192495

[31] Eddy SR (2009) A new generation of homology search tools based on probabilistic inference. Genome Informatics 23: 205–211. 20180275

[32] Harris MA, Clark J, Ireland A, Lomax J, Ashburner M, et al (2004) The Gene Ontology (GO) database and informatics resource. Nucleic Acids Research 32: D258–261. 14681407 10.1093/nar/gkh036 PMC308770

[33] Huang DW, Sherman BT, Lempicki RA (2008) Systematic and integrative analysis of large gene lists using DAVID bioinformatics resurces. Nature Protocols 4: 44–57. 10.1038/nprot.2008.211 19131956

[34] Westerlund B, Korhonen TK (2006) Bacterial proteins binding to the mammalian extracellular matrix. Molecular Microbiology 9: 687–694. 10.1111/j.1365-2958.1993.tb01729.x 7901732

[35] Vercellotti GM, McCarthy JB, Lindholm P, Peterson PK, Jacob HS, et al (1985) Extracellular matrix proteins (fibronectin, laminin, and type IV collagen) bind and aggregate bacteria. American Journal of Pathology 120: 13–21. 4014440 PMC1887975

[36] Nelson JA (1924) Morphology of the honeybee larva. Journal of Agricultural Research 28: 1167–1213.

[37] McQuilton P, St Pierre SE, Thurmond J, Consortium F (2012) FlyBase 101 - the basics of navigating FlyBase. Nucleic Acids Research 40: D706–D714. 22127867 10.1093/nar/gkr1030 PMC3245098

[38] Robertson C (1936) The metamorphosis of *Drosophila melanogaster*, including an accurately timed account of the principal morphological changes. Journal of Morphology 59: 351–399.

[39] Winston M (1991) Biology of the honey bee. Cambridge, Mass.: Harvard University Press.

[40] Gallot A, Rispe C, Leterme N, Gauthier J-P, Jaubert-Possamai S, et al (2010) Cuticular proteins and seasonal photoperiodism in aphids. Insect Biochemistry and Molecular Biology 40: 235–240. 20018241 10.1016/j.ibmb.2009.12.001

[41] Emerson KJ, Bradshaw WE, Holzapfel CM (2010) Microarrays reveal early transcriptional events during the termination of larval diapause in natural populations of the mosquito, *Wyeomyia smithii* . PLoS One 5: e9574. 20221437 10.1371/journal.pone.0009574 PMC2832704

[42] Vontas J, David J-P, Nikou D, Hemingway J, Christophides G, et al (2007) Transcriptional analysis of insecticide resistance in *Anopheles stephensi* using cross-species microarray hybridization. Insect Molecular Biology 16: 315–324. 17433071 10.1111/j.1365-2583.2007.00728.x

[43] Togawa T, Dunn WA, Emmons AC, Nagao J, Willis JH (2008) Developmental expression patterns of cuticular protein genes with the R&R Consensus from *Anopheles gambiae* . Insect Biochemistry and Molecular Biology 38: 508–519. 18405829 10.1016/j.ibmb.2007.12.008 PMC2416445

[44] Cornman RS, Willis JH (2009) Annotation and analysis of low-complexity protein families of *Anopheles gambiae* that are associated with cuticle. Insect Molecular Biology 18: 607–622. 19754739 10.1111/j.1365-2583.2009.00902.x PMC3701952

[45] Anders S, Huber W Differential expression analysis for sequence count data. Genome Biology 11: R106. 20979621 10.1186/gb-2010-11-10-r106 PMC3218662

[46] Mao W, Schuler MA, Berenbaum MR (2011) CYP9Q-mediated detoxification of acaricides in the honey bee (*Apis mellifera*). Proc Natl Acad Sci U S A 108: 12657–12662. 21775671 10.1073/pnas.1109535108 PMC3150950

[47] Gygi SP, Rochon Y, Franza BR, Aebersold R (1999) Correlation between protein and mRNA abundance in yeast. Molecular Cellular Biology 19: 1720–1730. 10022859 10.1128/mcb.19.3.1720 PMC83965

